# Syndromic and biological screening for sexually transmitted infections in female sex workers in Côte d’Ivoire: the ANRS 12381 PRINCESSE cohort study

**DOI:** 10.3389/fpubh.2025.1535122

**Published:** 2025-03-12

**Authors:** Marcellin N’zebo Nouaman, Patrick Ahuatchi Coffie, Aline A. Agoua, Clémence Zébago, Hervé Z. Dao, Evelyne Kissi, Severin Lenaud, Mian Anatole, Valentine Becquet, Mélanie Plazy, François Dabis, Serge Eholie, Joseph Larmarange

**Affiliations:** ^1^Programme PAC-CI, Site ANRS Côte d'Ivoire, Abidjan, Côte d'Ivoire; ^2^Département de Santé Publique et d’Odontologie Légale, UFR d’Odonto-Stomatologie, Université Félix Houphouët-Boigny, Abidjan, Côte d'Ivoire; ^3^Bordeaux Population Health Research Center, Université de Bordeaux, Inserm, IRD, Bordeaux, France; ^4^Département de Dermatologie et Infectiologie, UFR des Sciences Médicales, Université Félix Houphouët Boigny, Abidjan, Côte d'Ivoire; ^5^ONG Aprosam, San Pedro, Côte d'Ivoire; ^6^Ined, Paris, France; ^7^Ceped, Université Paris Cité, IRD, Université Sorbonne Paris Nord, Inserm, Paris, France

**Keywords:** sexually transmitted infections, female sex workers, key population, syndromic STI, biological STIs screening, Sub-Saharan Africa

## Abstract

**Background:**

Female sex workers (FSWs) are at high risk of contracting STIs, in particular in Sub-Saharan Africa. The implementation of oral HIV pre-exposure prophylaxis provided an opportunity to draw attention to the sexual health needs of FSWs. Innovative strategies to screen for and reduce the burden of STIs is thus a priority. This study describes STI screening among FSWs enrolled in the PRINCESSE project in Côte d’Ivoire.

**Methods:**

The PRINCESSE project (2019–2023) was an interventional cohort of FSWs ≥18 years, evaluating a comprehensive, community-based sexual and reproductive health care package, including the management of STIs, offered through mobile clinics operating on prostitution sites in San Pedro area. HIV testing and syndromic STI testing were offered at baseline and every 3 months. Biological testing of *Chlamydia trachomatis* (CT) and *Neisseria gonorrhoeae* (NG) was offered annually. Clinical forms included sociodemographic, behavioral and sex-work-related characteristics. We describe baseline characteristics, coverage of clinical examination, and vaginal, anal swab collection. Social, behavioral and sex work-related factors associated with an STI syndromic diagnosis were explored. A multivariable logistic regression model was used to identify factors associated with diagnosing a symptomatic STI.

**Results:**

489 FSWs were included in the PRINCESSE cohort. Median age was 29 years (24–35 years), 28.6% had had sex without a condom in the last 7 days. The prevalence of HIV at baseline was 10.5%. Only one case of HIV seroconversion was observed during the project. The most frequent symptom was ano-vaginal discharge (19.1%). The prevalence of STI based on clinical symptoms was 26.6%. The proportion of vaginal swab samples for which the PCR result was positive was 8.0% for CT and 4.0% for NG. Only age remained significantly associated with diagnosing a symptomatic STI in the multivariable analysis.

**Conclusion:**

This study revealed a high prevalence of HIV and STIs, similar to national estimates among FSWs enrolled in a sexual health cohort. Screening for these generically asymptomatic bacterial STIs must be combined with the syndromic approach used in key populations, especially with the introduction of new PrEP programs, to reduce the exposure of individuals in these populations to STIs.

## Introduction

Sexually transmitted infections (STIs) are frequent causes of severe illness and death, particularly among women, and affect the physical, mental and social well-being of individuals worldwide (1). Sub-Saharan Africa remains particularly affected, accounting for more than 40% of the global burden of STIs (2–4).

In Africa, women are generally more exposed to STIs than men are. In particular, female sex workers (FSWs) are at high risk of contracting STIs because of multiple sexual partners, condomless sex and addictive substance use (5, 6). The most recent estimates indicated an overall STI prevalence ranging from 4.2% for *Neisseria gonorrhoeae* (NG) to 32.9% for human papillomavirus (HPV) among FSWs in Togo in 2017 (7, 8). A study carried out in 2012 among FSWs in Abidjan, Côte d’Ivoire, reported an NG prevalence of 12.8% (9). Recently, the ANRS 12361 PrEP-CI study reported a high prevalence of STIs self-reported by FSWs in the regions of San Pedro and Abidjan in the last 12 months (78.8 and 55.2%, respectively) (10).

In most Western African countries, including Côte d’Ivoire, the management of STIs is based on a syndromic approach with screening for clinical signs and treatment via national drug kits based on a national algorithm, following 1984 World Health Organization (WHO) recommendations (11). In the absence of biological screening, missed opportunities for the diagnosis and treatment of STIs exist, with severe repercussions for women’s health (1). Since 2016, and with an update in 2021, the WHO has recommended strengthening national laboratory capacity through quality assurance and the introduction of point-of-care diagnostics to ensure the routine monitoring of STIs and antimicrobial resistance to NG (11, 12). Indeed, the early diagnosis of STIs, including those without symptoms, is the best opportunity for providing effective medical treatment and support and for preventing further transmission (11).

Since 2015, the implementation of oral HIV preexposure prophylaxis (PrEP) for populations at substantial risk of acquiring HIV, including the FSW population (13), has provided an opportunity to draw attention to the sexual health needs of key populations. Indeed, regular STI screening and treatment are crucial components of HIV PrEP initiation and follow-up (11). Identifying innovative strategies to screen for and reduce the burden of STIs in key populations is thus a priority in this context.

The ANRS 12381 PRINCESSE project was an interventional cohort study that was implemented at prostitution sites in the region of San Pedro in Côte d’Ivoire and offered a comprehensive sexual and reproductive health package to FSWs. This paper describes STI screening at baseline, including syndromic screening and biological testing, providing coverage and prevalence estimates as well as associated factors.

## Materials and methods

### Study setting

The PRINCESSE study was conducted in San Pedro and its surrounding areas. This region is home to agricultural enterprises, including coffee and cocoa plantations in urban zones and palm oil and hevea plantations in rural areas. Seasonal labor migration is high in this area and, consequently, seasonal migration of sex workers. San Pedro’s harbor is the world’s largest cocoa bean exporter and one of the largest harbors in West Africa.

The PRINCESSE study was developed in collaboration with Aprosam, a community-based organization that provides HIV prevention and testing services directly at prostitution sites through outreach activities and HIV and sexual and reproductive health care services to FSWs through a community clinic based in San Pedro. In a preparatory study conducted from 2016 to 2017, we estimated the incidence of HIV among FSWs reached by Aprosam to be 3.2 cases per 100 person-years (14).

### PRINCESSE cohort

The ANRS 12381 PRINCESSE project involved a single-arm interventional cohort of FSWs. The main objective was to develop, document, and analyze a community-based healthcare package of comprehensive sexual and reproductive health services that combined testing and prevention tools, including PrEP, immediate HIV treatment, and hepatitis B virus management. The full protocol is available online in French,^1^ and a summary has been published in English (15).

The PRINCESSE intervention package was delivered directly at prostitution sites via a mobile clinic that visited 10 sites (five urban sites in the city of San Pedro and five rural sites) every 2 weeks. The participants could also receive care in the fixed clinic operated by Aprosam in the city of San Pedro. The mobile clinic included a consultation room, a laboratory and a counseling room. The operational team comprised a physician, a laboratory technician, a social worker, two FSW peer educators, an interviewer, and a driver. Aprosam’s peer educators carried out community mobilization at the prostitution sites.

All women aged 18 years or older who self-reported being sex workers and wished to enroll for regular clinical follow-up were eligible for inclusion, regardless of their HIV status.

### HIV screening

HIV testing (including pre- and posttest counseling) was offered at baseline and every 3 months thereafter for those who previously tested negative. In accordance with the national algorithm, the tests used to screen for HIV infection were two rapid tests. We first used a rapid test (Determine®, Alere, sensitivity: 100%; specificity: 98.9%). If the test result was positive, HIV infection was confirmed with a second rapid test (Stat-pack®, sensitivity: 99.5%; specificity: 100%). In the case of a discrepancy, the Biolane® test was performed in the laboratory to determine the final result.

Newly diagnosed HIV-positive women were asked to initiate antiretroviral treatment as soon as possible, according to national guidelines. Women who tested negative for HIV were offered oral PrEP if they were interested.

### Syndromic STI screening

At baseline and every 3 months thereafter, participants were offered a clinical examination to identify STI symptoms, such as anovaginal ulcerations, anovaginal discharge, lower abdominal pain and cervical inflammation. We used a national algorithm to propose an STI treatment (national STI drug kits) based on these clinical signs and in the absence of biological diagnosis (16). In the event of complications, participants were referred to a level-2 center (Regional Hospital Centre) for care and support (16).

### Biological screening of *Chlamydia trachomatis* and *Neisseria gonorrhoeae*

At baseline and then annually, if the participant agreed, vaginal and anal swab samples were collected by the physician. The samples were kept at the Aprosam fixed clinic laboratory until they were sent to the CeDReS laboratory in Abidjan (the first isocertified laboratory in West Africa), where they were tested for *Chlamydia trachomatis* (CT) and NG via polymerase chain reaction (PCR) analysis. When a bacterial STI was isolated after PCR analysis, the participant was contacted by phone by the medical team to schedule an appointment to receive adequate STI treatment.

### Additional data collection

Clinical forms were completed during each clinic visit by the project staff and included sociodemographic, behavioral and sex-work-related characteristics.

### Data analysis

We used descriptive statistics to report the following: (i) baseline characteristics of the included participants; (ii) results of HIV testing at baseline and occurrence of HIV seroconversion; (iii) coverage of clinical examination, clinical signs, STI syndromic diagnosis and drug prescription at baseline; (iv) coverage of vaginal and anal swab collection and the results of CT and NG PCR analysis at baseline; and (v) the presence/absence of clinical signs among women with a positive PCR result.

Social, behavioral and sex work-related factors associated with an STI syndromic diagnosis at baseline were explored via Pearson’s chi-square tests for categorical variables, with all expected cell counts equal to or greater than 5, and via Fisher’s exact tests for categorical variables, with any expected cell count less than 5 (bivariable analysis). A multivariable binomial logistic regression was computed and reduced via a backwards stepwise approach by minimizing the Akaike information criterion (AIC).

All analyses were performed via R software (version 4.3.2) and the gtsummary package (17).

### Ethical aspects

The PRINCESSE protocol version 1.0 was submitted to the Comité National d’Ethique des Sciences de la vie et de la santé de la Côte d’Ivoire (CNESVS) in November 2018. The second version, including the changes requested by the CNESVS, was reviewed and approved on 4 March 2019 (ref: 152–18/MSHP/CNESVS-km). The third version, which included in-depth interviews conducted by phone, was approved on 15 March 2021. The fourth version, in which a biological component was added, was approved on 18 May 2021. The fifth version, which extended the cohort study until June 2023 and included an additional nested qualitative study, was approved on 29 April 2024. This ethics approval covered all the study sites.

The PRINCESSE project was registered on the Clinicaltrial.gov website (NCT03985085).

## Results

### Sociodemographic, behavioral and sex-work-related characteristics of the included participants

From 26 November 2019 to 30 June 2023, 489 FSWs were included in the PRINCESSE cohort. The median age was 29 years (interquartile range [IQR] = 24–35 years), 55.9% of the participants were Ivorian citizens, 82.1% were not living with a partner, 35.8% had never been to school, and 28.6% had had sex without a condom in the last 7 days (Table 1).

**Table 1:** Sociodemographic, behavioral and sex-work-related characteristics at inclusion of the 489 female sex workers included in the PRINCESSE Project, San Pedro, Côte d’Ivoire, 2019–2023.

Characteristics at inclusion	% (*n*)
Age group	
35 years old or older	26.2 (128)
25–34 years old	48.1 (235)
18–24 years old	25.8 (126)
Education level	
Never went to school	35.8 (174)
Primary	30.7 (149)
Secondary or more	33.5 (163)
Not reported	3
Number of clients on the last day of work	
4 clients or less	55.1 (236)
5 clients or more	44.9 (192)
Not reported	61
Nationality	
Ivoirian	55.9 (272)
other nationality	44.1 (215)
Not reported	2
Lives with a partner	
No	82.1 (395)
Yes	17.9 (86)
Not reported	8
Sex work as the main activity	
No	46.4 (227)
Yes	53.6 (262)
Usual price for sex work	
≤1,500 CFA (2 USD)	43.7 (198)
>1,500 CFA	56.3 (255)
Not reported	36
Condomless sex in the last 7 days	
No	71.4 (345)
Yes	28.6 (138)
Not reported	6
Experience in sex work	
≤ 3 years	66.3 (319)
> 3 years	33.7 (162)
Not reported	8
Frequency of sex work	
Almost everyday	93.9 (446)
At least once per week	5.5 (26)
At least once per month	0.6 (3)
Not reported	14
Period of enrolment	
<2020	61.8 (302)
≥2020	38.2 (187)
Site of enrolment	
Urban site	50.9 (249)
Rural site	49.1 (240)
Place of sex work (several locations possible)	
In hotels	68.5 (335)
In brothels	52.8 (258)
In other places (at home, bar/maquis)	30.9 (151)

### HIV infection

Among the 489 women included in the study, 478 (97.8%) were screened for HIV at the inclusion visit, and 50 tested positive, resulting in a prevalence of 10.5% [95% confidence interval (CI): 7.9–13.6%].

Only one case of HIV seroconversion was detected during the project: this woman tested negative for HIV at inclusion but was retested 2 weeks later during the PrEP initiation visit and had a positive result. She was likely in the initial stage of infection at the time of inclusion.

### Clinical examination, clinical signs, STI syndromic diagnosis and drug prescription at baseline

Among the 489 FSWs included in the cohort, 440 (90.0%) underwent a clinical examination for STI diagnosis (Table 2). The most frequent symptom was anovaginal discharge (*n* = 84, 19.1%), followed by lower abdominal pain (*n* = 26, 5.9%), cervical inflammation (*n* = 15, 3.4%), and anovaginal ulcers (*n* = 12, 2.7%). For 117 women, the physician diagnosed an STI on the basis of clinical symptoms, resulting in a prevalence of 26.6% [22.6–31.0]. Among these women, 90 (76.9%) received a prescription for an STI drug kit.

**Table 2:** Clinical examination, clinical signs, STI syndromic diagnosis and drug prescription at baseline among the 489 FSWs included in the PRINCESSE cohort.

	% (n/N)	95% Confidence interval
Clinical examination performed	**90.0 (440/489)**	**86.9–92.4**
Clinical signs		
Anovaginal ulcers	2.7 (12/440)	1.48–4.85
Anovaginal discharge	19.1 (84/440)	15.6–23.1
Cervical inflammation (cervicitis)	3.4 (15/440)	1.99–5.69
Lower abdominal pain	5.9 (26/440)	3.97–8.65
STI diagnosed by the physician based on symptoms	**26.6 (117/440)**	**22.6–31.0**
Prescription of STI drug kits	**76.9 (90/117)**	**68.0–84.0**

Bolded elements in the tables to highlight a few key messages.

### Vaginal and anal swab collection and PCR results for *Chlamydia trachomatis* and *Neisseria gonorrhoeae* at baseline

Among the 489 FSWs included, 349 (71.4%) vaginal and 337 (68.9%) anal swab samples were obtained. There were various reasons why the swabs of some participants were not collected, including the presence of menstrual blood (123, 47.0%), participant refusal (101, 38.0%), or the presence of an STI with clinical signs such as pain or ulceration (36, 14.0%).

Among the 349 participants for whom a vaginal swab sample was collected, the laboratory did not have enough biological material to perform PCR for CT in 6 cases and for NG in one case. In addition, some PCR results were undetermined (*n* = 9, 2.6%). The proportion of vaginal swab samples for which the PCR result was positive was 8.0% [5.5–11.5] for CT and 4.0% [2.3–6.8] for NG (Table 3).

**Table 3:** Vaginal and anal swab collection and PCR results for *Chlamydia trachomatis* and *Neisseria gonorrhoeae* at baseline among the 489 FSWs in the PRINCESSE cohort.

	% (n/N)	95% Confidence Interval
Vaginal swab collected	**71.4 (349/489)**	**67.1–75.3**
*Chlamydia Trachomatis*		
Negative PCR result	87.7 (306/349)	83.7–90.8
Positive PCR result	8.0 (28/349)	5.5–11.5
Undetermined PCR result	2.6 (9/349)	1.3–5.0
PCR not performed (insufficient sample)	1.7 (6/349)	0.7–3.9
*Neisseria Gonorrhoeae*		
Negative PCR result	93.1 (325/349)	89.8–95.5
Positive PCR result	4.0 (14/349)	2.3–6.8
Undetermined PCR result	2.6 (9/349)	1.3–5.0
PCR not performed (insufficient sample)	0.3 (1/349)	0.01–1.8
Anal swab collected	**68.9 (337/489)**	**64.6–73.0**
*Chlamydia Trachomatis*		
Negative PCR result	74.2 (250/337)	69.1–78.7
Positive PCR result	3.3 (11/337)	1.7–5.9
Undetermined PCR result	22.6 (76/337)	18.3–27.5
PCR not performed (insufficient sample)	0.0 (0/337)	0.0–1.4
*Neisseria Gonorrhoeae*		
Negative PCR result	76.3 (257/337)	71.3–80.6
Positive PCR result	0.9 (3/337)	0.2–2.8
Undetermined PCR result	22.6 (76/337)	18.3–27.5
PCR not performed (insufficient sample)	0.3 (1/337)	0.02–1.9

Bolded elements in the tables to highlight a few key messages.

Among the 337 participants for whom a vaginal swab sample was collected, there was not enough material present for the laboratory to perform PCR for NG in one case. The number of participants with undetermined PCR results was relatively high for both CT and NG (*n* = 76, 22.6%). The number of anal swab samples for which the PCR result was positive was 3.3% [1.7–5.9] for CT and 0.9% [0.2–2.8] for NG.

### Clinical signs among women with a positive PCR result

Most women with an STI diagnosed by PCR were not diagnosed with a syndromic STI at the baseline visit. Among the 28 women with vaginal CT diagnosed by PCR, only three (10.7%) were diagnosed with a syndromic STI by the physician during the clinical examination at inclusion (Table 4). Similarly, the number of women diagnosed with syndromic STIs were as follows: 1/14 (7.1%) women were diagnosed with vaginal NG, 1/11 (9.1%) women were diagnosed with anal CT, and 0/3 (0.0%) women were diagnosed anal NG.

**Table 4:** Clinical signs among women with a positive PCR result at baseline.

	Positive PCR result			
	Vaginal CT % (n/N)	Vaginal NG % (n/N)	Anal CT % (n/N)	Anal NG % (n/N)
Sign observed at clinical examination				
Anovaginal ulcer	0.0 (0/28)	0.0 (0/14)	0.0 (0/11)	0.0 (0/3)
Anovaginal discharge	7.1 (2/28)	7.1 (1/14)	9.1 (1/11)	0.0 (0/3)
Cervical inflammation (cervicitis)	0.0 (0/28)	0.0 (0/14)	0.0 (0/11)	0.0 (0/3)
Lower abdominal pain	3.6 (1/28)	7.1 (1/14)	9.1 (1/11)	0.0 (0/3)
STI diagnosed by the physician based on symptoms	**10.7 (3/28)**	**7.1 (1/14)**	**9.1 (1/11)**	**0.0 (0/3)**

CT, *Chlamydia trachomatis*, NG, *Neisseria gonorrhoeae*. Bolded elements in the tables to highlight a few key messages.

The most common associated clinical symptom was anovaginal discharge.

### Factors associated with syndromic STIs at baseline

Factors associated with syndromic STIs were investigated among FSWs examined at baseline and with syndromic STI results recorded in the consultation file.

According to the bivariable analysis, FSWs were more likely to have syndromic STIs if they were < 35 years old (*p* = 0.003) or worked in hotels (0.016) (Table 5). In the multivariable analysis, only age remained significantly associated with the diagnosis of a symptomatic STI by the physician during the baseline clinical examination at the inclusion visit. FSWs aged 25–35 years and over 35 years were less likely to have STIs than younger FSWs were (18–24 years), with odds ratios of 1.96 [95% CI: 1.3–3.3] and 2.38 [1.4–5.0], respectively (Table 5).

**Table 5:** Diagnosis of a syndromic STI by the physician and associated factors among the 435 FSWs in the PRINCESSE cohort who were clinically examined and notified in the register during their baseline visit.

	Diagnosed with a syndromic STI at the baseline examination		Bivariable analysis	Multivariable analysis		
	n/N	%	*p* value	aOR	95% CI	*p* value
Age group			0.003			
35 years old or older	24/117	20.5				
25–34 years old	51/214	23.8		1.96	1.3–3.3	0.010
18–24 years old	41/104	39.4		2.38	1.4–5	0.006
Education level			0.3			
Never went to school	45/151	29.8				
Primary	29/133	21.8				
Secondary or more	42/149	28.2				
Number of clients on the last day of work			>0.9			
4 clients or less	56/216	25.9				
5 clients or more	43/169	25.4				
Not reported	17					
Nationality			0.2			
Ivoirian	69/239	28.9				
Other nationality	46/194	23.7		0.70	0.4–1.1	0.13
Not reported	1					
Lives with a partner			0.057			0.066
No	100/351	28.5				
Yes	14/78	17.9		0.55	0.3–1.0	
Not reported	2					
Sex work as the main activity			0.5			0.5
No	50/200	25.0				
Yes	66/235	28.1		1.18	0.7–1.9	
Usual price for sex work			0.7			
1,500 CFA or less (2 USD)	47/175	26.9				
1,500 CFA or more	58/230	25.2				
Not reported	11					
Condomless sex in the last 7 days			0.8			
No	82/313	26.2				
Yes	33/120	27.5				
Not reported	1					
Experience in sex work			0.8			
3 years or less	76/283	26.9				
4 years or more	38/147	25.8				
Not reported	2					
HIV status at baseline			0.5			
Negative	105/377	27.8				
Positive	11/48	22.9				
Frequency of sexual activity			0.3			
Everyday	104/397	26.2				
At least once per week	7/24	29.2				
At least once per month	2/3	66.7				
Not reported	3					
Period of enrolment			0.8			
<2020	69/262	26.3				
≥2020	47/173	27.2				
Site of enrolment			0.14			
Urban site	48/214	22.4				
Rural site	59/206	28.6				
Not reported	9					
Sex work in hotels			0.11			
Yes	72/296	24.3				
No	44/139	31.6				
Sex work in brothels			>0.9			
Yes	61/230	26.5				
No	55/205	26.8				
Other places of activity (at home, bar/maquis)			0.2			
Yes	41/131	31.3				
No	75/304	24.7				

aOR, adjusted odds ratio; CI, confidence interval; *p* value, *p* < 0.05 was considered statistically significant.

## Discussion

At baseline, among the 489 FSWs included in the PRINCESSE cohort, almost all were tested for HIV, and most of them were clinically examined for STIs. The prevalence of STIs remained relatively high in this population: 10.5% of the women were HIV positive, and 26.6% were diagnosed with syndromic STIs on the basis of clinical symptoms.

As part of the overall package offered by the PRINCESSE project, all participants were offered a clinical medical examination, including a gynecological examination and the collection of anovaginal swab samples for biological screening. However, approximately 10% of the participants did not have STIs at clinical examination, and 30% did not agree to have ano-vaginal swab samples taken. We were unable to systematically document the reasons why clinical examinations were not carried out, but the health workers frequently reported a lack of time. On the other hand, several reasons were given by the FSWs who had received a gynecological examination but for whom anovaginal swab samples had not been taken. The reasons included the presence of menstrual blood, time constraints (especially when clients were waiting), pain or ulcerations, or a lack of comfort. Studies conducted in Australia and England also reported several obstacles to the collection of conventional anovaginal swab samples by medical professionals, particularly the sensation of discomfort and pain, the country’s culture, fear of the result being announced, a history of trauma or sexual abuse and, above all, the lack of time synonymous with the loss of money (18–22). This rate of collection of anovaginal swabs remains suboptimal for ensuring widespread coverage of asymptomatic STI screening and treatment among FSWs.

To contribute to the widespread coverage of STI screening among FSWs, the option of self-testing could be proposed for this population. In New Zealand, women prefer self-sampling to conventional sampling, and the results are as reliable as those of PCR tests (23). Self-diagnosis of STIs removes barriers to screening while increasing participation and acceptability and maintaining the quality of care (24–27). We needed to anticipate certain operational difficulties in our interventions to ensure widespread coverage of screening and optimal care for FSWs. In fact, 23.1% of the participants in our study did not receive treatment despite the presence of a syndromic STI diagnosed by the medical doctor.

Logistical and operational difficulties made the management of STIs in our intervention package somewhat complex, particularly the time taken to deliver results and shortages of inputs.

The anovaginal swab samples were preserved in the laboratory of the partner NGO at the study site in San Pedro before being transported to the CeDRes (study reference laboratory) in Abidjan (350 km from San Pedro) for PCR analysis. This distance meant that swab samples could not be transported very quickly, as many samples had to be taken before they could be transported. The results of the examinations arrived late, after approximately 3–4 weeks. Under these circumstances, FSWs with positive PCR results were not rescheduled for appropriate management because of the late availability of results (28). As a result, follow-up can be impeded, and care or treatment can be incomplete, as has already been reported (29). Because of their extreme mobility, it was not only very difficult to see these women again, even in the event of a positive result, but also complicated to schedule an appointment for appropriate follow-up. It was also difficult for these women to visit the fixed clinic to receive treatment if needed because of their financial difficulties. Given their precarious situations, the medical team offered the women appointments at the fixed clinic for follow-up and reimbursed their transportation costs. This proposal was not followed up (15, 30). Additionally, the frequent shortages of drug kits mean that not all women diagnosed with an STI can be treated (28).

It is therefore essential to move toward point-of-care tools and have essential medicines available at outposts for immediate treatment after diagnosis (29). These point-of-care tools have already been evaluated and shown to be effective in detecting CT, NG and *Trichomonas vaginalis* (31). This would be an opportunity not only to screen many particularly mobile FSWs but also to reduce the anxiety and distress associated with waiting for the results of conventional PCR tests.

The estimated HIV prevalence of 10.5% (1.9–13.6) at inclusion in the PRINCESSE cohort was relatively high compared with the national estimates. In Côte d’Ivoire, the HIV prevalence among women in the general population aged between 15 and 49 years and among FSWs was estimated at 2.6 and 4.8% in 2023, respectively, according to UNAIDS 2024 country data (32). There are specific regions, such as San Pedro, where the epidemic remains concentrated, with incidences higher than those in Abidjan (3.3% in San Pero vs. 1.6% in Abidjan) (14). HIV is still concentrated in certain areas of the country and in groups in precarious situations and with difficult living conditions, such as FSWs in San Pedro. The link between job insecurity, the working conditions of FSWs and the risk of exposure to HIV has already been shown in a previous study of this population (14). Despite national HIV prevention efforts, the prevalence of HIV remains stable and concentrated in certain groups. Current conventional interventions to combat HIV infection need to be implemented in remote, hard-to-reach areas such as San Pedro and surrounding villages.

The prevalence of syndromic STIs among FSWs included in the PRINCESSE project (26.6%; 22.6–31.0) corroborates the findings of a study conducted among women in the general population in Côte d’Ivoire in 2019 (30%) (33). While a higher prevalence of STIs in the FSW population than in the general population was expected due to sex work activity, the prevalence remains similar despite the intense prevention activities provided through various intervention programs for this target group.

In other West African countries, the prevalence of STIs among FSW varies from 17.2% in Togo (34) to 19.7% in Guinea Bissau (35), even reaching 35.1% in Mali (36). This variation in the prevalence of STIs could be due to different sociobehavioural and structural factors of exposure to STIs, which may vary from one context to another. These factors include violence, multiple sexual partnerships, stigmatization and criminalisation, as well as barriers to accessing healthcare (37–40). Thus, there is a need to develop programs with the target populations to identify the factors for effective STI reduction interventions.

The clinical signs most frequently associated with syndromic STIs diagnosed by the doctor were anovaginal discharge and lower abdominal pain (71.8 and 22.2%, respectively). In a study conducted in Togo in 2022, vaginal discharge was also the most frequent clinical sign, with an estimated prevalence of 67.2% (34).

In Côte d’Ivoire, according to data from the National AIDS Program in 2019, the most frequent clinical symptoms of STIs were lower abdominal pain and vaginal discharge in 70% of the patients diagnosed with STIs (33). These clinical signs are characteristic of a curable parasitic infection with *Trichomonas vaginalis* in women, which is widely described in the literature (41), with serious complications that can lead to adverse pregnancy outcomes, premature delivery, low birth weight, infertility and cervical cancer (42). As our results show that some FSWs have syndromic STIs, it would therefore be appropriate to include syndromic screening in the sexual and reproductive health services for this particular group and thus contribute to these individuals’ well-being.

The estimated prevalence of curable vaginal bacterial STIs in our study was 8.0% (5.5–11.5) for CT and 4% (2.3–6.8) for NG, similar to the 2012 figures among FSWs visiting dedicated medical clinics in Abidjan and San Pedro (7.9 and 5.5%, respectively) (43). PCR detected CT in 3.3% (1.7–5.9%) of the anal swab samples and NG in 0.9% (0.2–2.8%) of the samples. While a higher prevalence was expected, the prevalence of curable bacterial STIs has remained stable since 2012.

Data on the prevalence of curable STIs have been reported in other areas of the region, such as Cotonou, Benin, where the respective prevalence rates of CT and NG were 4.1 and 2.8%, respectively, among FSWs in 2018 (44), and Burkina Faso, where the prevalence rates of CT and NG were 11.5 and 13.7%, respectively (45). In 2022, in Nairobi (Kenya), the prevalence of NG on vaginal swabs was estimated at 6.3% in FSWs (46).

The prevalence of CT and NG observed in our study could be influenced by the sampling and biological analysis techniques used. In fact, 4.3% of the vaginal swab samples taken in our study for the purpose of testing for CT did not yield conclusive results, either because the sample was insufficient for the test or because the result of the test was undetermined. This proportion of undetermined results was even greater (22.6%) for anal swab samples. Although the literature does not mention that anovaginal sampling techniques are operator dependent, this is a hypothesis that should be considered.

The prevalence of CT and NG could also be influenced by behavioral habits such as self-medication with both modern and traditional medicines and the fact that most FSWs in this population reported systematic condom use. The results obtained in 2016 in this same population revealed a high rate of condom use during sexual encounters and the practice of self-medication by FSWs rather than consultation with healthcare professionals (10).

Genital infections caused by CT are the most widespread infections in the world and are mainly asymptomatic (47). Most of the cases of CT and NG infection detected in our study were not associated with clinical signs. Only 10.7 and 7.1% of the CT and NG cases, respectively, were associated with clinical signs. The main clinical sign was anovaginal discharge (7.1% for both CT and NG). The presence of anovaginal discharge in cases of CT infection has already been described (48).

Similarly, in the Netherlands in 2022, a study of 524 women at a specialist STI clinic revealed that a viable CT viral load was independently associated with genital symptoms, particularly vaginal discharge (49). Notably, vaginal discharge is often associated with parasitic and mycotic infections caused by *Trichomonas vaginalis* and *Candida albicans* (41, 50). Vaginal discharge is therefore common in women with bacterial, mycotic and parasitic infections (50). This may reflect the fact that the relevance of screening and treatment of asymptomatic STIs such as CT for public health reasons is controversial (51). The debate centers on whether the infection is highly prevalent in the population concerned. Additionally, anal infections in women, which are often asymptomatic and can lead to complications in terms of sexual and reproductive health, remain speculative and unquantified (51). Notably, in our study, the PCR results were undetermined for approximately 30% of the anal swab samples (vs. 3% for the vaginal swab samples). This may lead to an underestimation of positive PCR results for anal swab samples. Additionally, most women with a positive PCR result did not present any visible symptoms at the time of clinical examination.

Screening for asymptomatic STIs therefore remains problematic in these circumstances. However, there is evidence that CT infections, although initially asymptomatic, lead to serious complications for women’s sexual and reproductive health services (48, 52, 53). These results suggest the need to combine syndromic STI screening and laboratory testing to detect and treat as many STIs as possible at an early stage in key populations, such as the sexually active FSW population, and thus contribute to the elimination of STIs by 2030, as recommended by the WHO.

Another approach could be periodic presumptive treatment if there are no suitable laboratories accessible at health posts to treat individuals with asymptomatic STIs. However, this approach raises the question of antimicrobial resistance, a real threat to public health (54, 55).

Our study has several limitations. The study sample was not completely representative of FSWs in San Pedro area. However, the FSWs were recruited through peer educators, who were able to enroll FSWs with a variety of profiles from different places, and this is one of the strong points of this study. Another limitation of this study is the high number of anovaginal swab samples taken without results, which could underestimate the respective prevalence rates of CT and NG. Nevertheless, the prevalence rates found remain similar to previous estimates in the country and elsewhere in West Africa.

Finally, the high drop-out rate in our study and the small number of participants did not allow us to identify the factors associated with syndromic STIs; indeed, we were unable to carry out regular follow-up of participants in the PRINCESSE cohort to assess the incidence of syndromic and bacterial STIs and identify the factors leading to the occurrence of these infections. Additionally, the women treated were not followed up to assess whether they were cured or whether the STIs had persisted. There is a need for further research to obtain more data on this issue.

## Conclusion

This study revealed a high prevalence of HIV and a high prevalence of STIs, similar to national estimates among FSWs enrolled in a sexual health cohort. Most STIs are syndromic, so there is a need to include syndromic screening in the sexual health and reproductive services offered to FSWs. In the face of these STIs, which are still common among key populations, strategies and/or efforts to reduce the burden of STIs on FSWs need to be revisited. Screening for these generically asymptomatic bacterial STIs must be combined with the syndromic approach used in key populations, especially with the introduction of new PrEP programs, to reduce the exposure of individuals in these populations to STIs and that of their partners and to improve their overall health.

## Data Availability

The datasets presented in this study can be found in online repositories. The names of the repository/repositories and accession number(s) can be found at: https://doi.org/10.23708/VZKQUF.

## Footnotes

^1^ https://hal.science/hal-04127364
